# Supplementary material for: Daytime admission is associated with higher 1-month survival for pediatric out-of-hospital cardiac arrest: Analysis of a nationwide multicenter observational study in Japan
Source: PLoS One. 2021 Feb 10;16(2):e0246896. doi: 10.1371/journal.pone.0246896 (PMC7875334; doi:10.1371/journal.pone.0246896)
Supplement: S1 Table — (DOCX) [file pone.0246896.s001.docx]

| S1 Table. Characteristics of patients according to admission time | | | | | | |
| --- | --- | --- | --- | --- | --- | --- |
| characteristics | **daytime admission**  **(9:00 a.m.-4:59 p.m.)**  **(n＝104）**  **frequency (%)/**  **median [interquartile range]** | | **nighttime admission**  **(5:00 p.m.-8:59 a.m.)**  **(n＝206)**  **frequency (%)/**  **median [interquartile range]** | | ***p* value** | |
| age group |  |  |  |  | 0.35 |  |
| infants (0-1 year) | 48 | (44%) | 91 | (44%) |  |  |
| young children (2-7 years) | 18 | (17%) | 28 | (14%) |  |  |
| older children (8-12 years) | 12 | (12%) | 17 | (8%) |  |  |
| teenagers (13-17 years) | 26 | (25%) | 70 | (34%) |  |  |
| witnessed OHCA | 35 | (34%) | 62 | (30%) | 0.41 |  |
| OHCA with bystander CPR | 47 | (45%) | 108 | (52%) | 0.32 |  |
| layperson AED application | 6 | (6%) | 1 | (0.5%) | 0.003 |  |
| cardiac origin OHCA | 26 | (25%) | 68 | (33%) | 0.15 |  |
| initial rhythm (ventricular fibrillation or pulseless ventricular tachycardia) | 5 | (5%) | 8 | (4%) | 0.66 |  |
| time from call to EMS arrival at scene, min | 7 | [5-8] | 7 | [5-9] | 0.07 |  |
| time from call to EMS arrival at hospital, min | 30 | [23.8-36.5] | 29 | [23-37] | 0.55 |  |
| Implementation of therapeutic hypothermia | 11 | (11%) | 11 | (5%) | 0.09 |  |
| Implementation of extracorporeal membrane oxygenation | 3 | (3%) | 4 | (2%) | 0.60 |  |
