# Supplementary material for: Daytime admission is associated with higher 1-month survival for pediatric out-of-hospital cardiac arrest: Analysis of a nationwide multicenter observational study in Japan
Source: PLoS One. 2021 Feb 10;16(2):e0246896. doi: 10.1371/journal.pone.0246896 (PMC7875334; doi:10.1371/journal.pone.0246896)
Supplement: S2 Table — (DOCX) [file pone.0246896.s002.docx]

| S2 Table. Characteristics of patients treated in the hospital with and without a PICU | | | | | |
| --- | --- | --- | --- | --- | --- |
| characteristics | **Treated in the hospital with a PICU**  **(n＝31）**  **frequency (%)**  **median [interquartile range]** | | **Treated in the hospital without a PICU**  **(n＝279)**  **frequency (%)**  **median [interquartile range]** | | ***p* value** |
| age group |  |  |  |  | 0.59 |
| infants (0-1year) | 12 | (39%) | 127 | (46%) |  |
| young children (2-7years) | 7 | (5%) | 39 | (16%) |  |
| older children (8-12years) | 2 | (15%) | 27 | (10%) |  |
| teenagers (13-17years) | 10 | (36%) | 86 | (31%) |  |
| sex male | 22 | (71%) | 178 | (64%) | 0.43 |
| cardiac origin OHCA | 16 | (52%) | 78 | (28%) | 0.008 |
| witnessed OHCA | 7 | (5%) | 90 | (32%) | 0.21 |
| admission to pediatric high-volume institute | 28 | (90%) | 187 | (67%) | 0.008 |
| the emergency department had one or more specialist physicians of intensive care | 31 | (100%) | 260 | (93%) | 0.13 |
| the emergency department had one or more pediatricians | 31 | (100%) | 199 | (71%) | 0.001 |
| one or more pediatrician participated during the resuscitation | 19/23 | (61%) | 89/229 | (32%) | 0.000 |
| daytime admission (09:00-16:59) | 9 | (29%) | 95 | (34%) | 0.58 |
| time from call to EMS arrival at scene, min | 6 | [4-8.3] | 7 | [5-9] | 0.22 |
| time from call to EMS arrival at hospital, min | 29.5 | [23.8-33.5] | 30 | [23-37] | 0.70 |
| Implementation of therapeutic hypothermia | 1 | (3%) | 21 | (8%) | 0.33 |
| Implementation of extracorporeal membrane oxygenation | 0 | (0%) | 7 | (3%) | 0.48 |
