# Supplementary material for: Daytime admission is associated with higher 1-month survival for pediatric out-of-hospital cardiac arrest: Analysis of a nationwide multicenter observational study in Japan
Source: PLoS One. 2021 Feb 10;16(2):e0246896. doi: 10.1371/journal.pone.0246896 (PMC7875334; doi:10.1371/journal.pone.0246896)
Supplement: Attachment — Submitted filename: Response to reviewers02.docx [file pone.0246896.s004.docx]

To the Editor:

We thank you for all of your reviews and thorough comments on our manuscript.

We numbered your comments for convenience.

Please find our responses to each comment below, in which we believe we have addressed all the concerns.

In this letter, we use the number of lines from a clean copy of the revised manuscript.

Reviewer Comments

Reviewer #1: Thank you for your efforts. The manuscript has been improved. However. I still have the following concern about your manuscript.

**Comment 1-1**

MAJOR

The main findings of this study are

1. Daytime admission was related to 1-month survival (odds ratio, OR: 95% confidence interval, CI, 3.64: 1.23 - 10.80)

2. Cardiac origin cardiac arrest and witnessed arrest were also associated with higher 1-month

survival (OR: 95% CI, 3.92: 1.23 - 12.47, and 6.25: 1.98 - 19.74, respectively).

Thus, Tables 1 and 2 are main tables. I recommend that Tables 3 and 4 are numbered as supplemental tables. Also, detailed explanation for supplemental tables are unnecessary.

**Response 1-1**

Thank you for your comment. We numbered Tables 3 and 4 from the previous version as S1 and S2 Tables in the Supplementary Material and removed the detailed explanation from the Results and the Discussion section.

1) We deleted and amended below in the Results:

Line 212, p. 13- Line 224, p. 14

*Among the children with OHCA, 19/104 (18%) survived at 1 month after a daytime OHCA compared to 20/206 (10%) after nighttime arrest. The results suggested that the time of admission was related to the outcome of pediatric OHCA. We also compared the two groups, based on the time of admission. There were no significant group differences, exept for the proportion except for proportion of layperson AED applications (S1 Table), proportion of patients with witnessed OHCA, and proportion who received bystander CPR according to admission time. Additionally, there were no significant differences in the rations of cardiac origin OHCA and EMS response time.*

*The number of patients treated in hospitals with a PICU was 31. Among them, only one patient survived 1 month after cardiac arrest. While there was no significant difference, the 1-month survival of patients treated in hospitals without a PICU was 11%. We also compared patient characteristics between the two groups, based on whether the accepting hospital had a PICU or not. In patients treated in the hospital with a PICU, a significantly higher rate of cardiac origin OHCA, admission to a pediatric high-volume institute with the presence of pediatricians, and treatment by pediatricians were observed (S2 Table).*

2) We deleted below from the previous version in the Discussion:

*Perhaps unexpectedly the nominal one-month survival rate was 3% among patients treated in hospitals with a PICU versus 11% in hospitals without a PICU, (p = 0.10). Although this was not a statistically significant difference, we further examined factors that might have been associated with survival rates in hospitals with PICU versus those without. There was no difference in age group or gender, and there was no difference in the rate of witnessed cardiac arrest. The EMS response time also did not differ. There was a difference in the rate of cardiac origin OHCA. Although detailed diagnosis was unknown, those cases might have a history of heart disease that had already been known. In addition, patients sent to a hospital with PICU may have had a higher rate of having some underlying diseases.*

**Comment 1-2**

MINOR

Abstract Results

“Cardiac origin cardiac arrest and witnessed arrest were also associated with

higher 1-month survival” should be read as “OHCA of presumed cardiac etiology and witnessed OHCA were associated with higher 1-month survival.

**Response 1-2**

Thank you for your comment. We changed the sentence, following your comment.

Line 31- 32, p. 2

*OHCA of presumed cardiac etiology and witnessed OHCA were associated with higher 1-month survival.*

Reviewer #2:

**Comment 2-1**

Dear Authors,

Thank you for addressing all of my comments and suggestions, I feel that the manuscript has improved substantially.

However, I would suggest having another look at the English grammar and style of your manuscript, as there are still a few sentences and passages that read confusing (e.g., presence or absence of articles,... etc.). The best way to overcome this would be involving a native English speaker.

**Response 2-1**

Thank you for your comment. We utilized an English editing service to improve our manuscript. We attached the editing certification for it.
